# Lab on a single microbead: an ultrasensitive detection strategy enabling microRNA analysis at the single-molecule level†
†Electronic supplementary information (ESI) available. See DOI: 10.1039/c5sc02641e


**DOI:** 10.1039/c5sc02641e

**Published:** 2015-08-20

**Authors:** Xiaobo Zhang, Chenghui Liu, Lingbo Sun, Xinrui Duan, Zhengping Li

**Affiliations:** a Key Laboratory of Applied Surface and Colloid Chemistry , Ministry of Education , Key Laboratory of Analytical Chemistry for Life Science of Shaanxi Province , School of Chemistry and Chemical Engineering , Shaanxi Normal University , Xi'an 710062 , Shaanxi Province , P. R. China . Email: liuch@snnu.edu.cn ; Email: lzpbd@snnu.edu.cn

## Abstract

Detection of a single nucleic acid molecule is of great significance for both fundamental biochemistry studies and clinical diagnostics. By using microRNA (miRNA) as a model target, herein, we have developed a single-microbead-based sensing (SMBS) platform, which simply enables the detection of miRNA at the single-molecule level. In this strategy, an isothermal exponential amplification reaction (EXPAR) is rationally designed towards specific miRNAs and all products of the EXPAR are integrated onto a single microbead for signal amplification and fluorescence enrichment. This pushes the detection of miRNAs down to 1 aM in a 5 μL sample, corresponding to 3 copies of the miRNA molecule. This new strategy also affords high selectivity and it is capable of distinguishing among homologous miRNA family members even with a single-base difference. Due to its ultrahigh sensitivity and selectivity, the proposed SMBS platform has been successfully applied to the detection of miRNA extracted from a single cell.

## Abstract 2

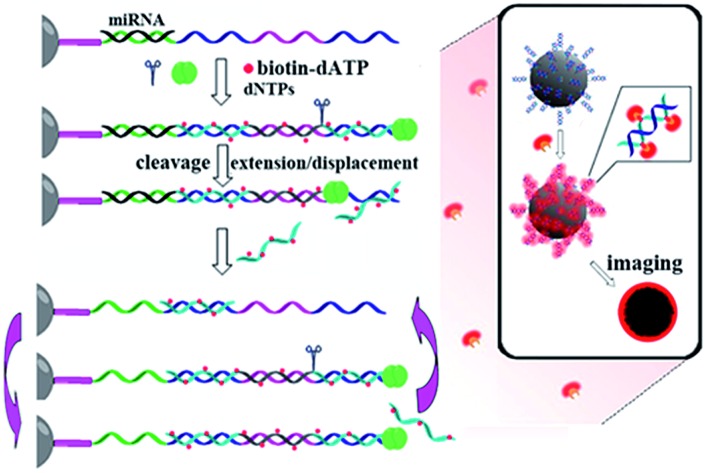
A single microbead-based sensing platform has been developed, which enables the detection of microRNA at the single-molecule level.

## Introduction

Gene expression, that is, genetic information encoded in DNA is transcribed to messenger RNA (mRNA) and then mRNA is translated to protein, is the central dogma of molecular biology in a living cell. Meanwhile, microRNA (miRNA) plays critical regulatory roles in the translation process. Since most genes and many RNA molecules of interest exist singly or in extremely low copy numbers in a cell, only by accurate detection of these single molecules can we fully understand their exact biofunction and behaviour.^1^–^3^ In this regard, the ultrasensitive detection of nucleic acid molecules at the single-molecule level is extremely important for both fundamental biochemistry studies and clinical diagnostics.

Over the past two decades, the rapid advances in single-molecule detection (SMD) techniques by the use of total internal reflection fluorescence microscopy (TIRFM), fluorescence correlation spectroscopy as well as some other complicatedly-modified fluorescence microscopes, have provided powerful tools for the fluorescence detection of individual, fluorescently-labeled macromolecules, including nucleic acids.^4^–^9^ However, these so-called SMD techniques do not mean that if there is only one target molecule in the sample it can be exactly detected because the SMD methods are generally limited by the extremely low interrogation volume allowed for laser scanning. So, at very low concentrations of the target, it is difficult to detect the individual fluorophores unless we know exactly where the individual molecules are and where to look under the microscope.^4^ As such, in fluorescence correlation spectroscopy or TIRFM experiments, individual molecules in the samples must be in the concentration range of pM to nM for them to be detected easily.^4^

Alternatively, homogeneous exponential nucleic acid amplification techniques (*e.g.* PCR and some isothermal exponential amplification protocols), which can replicate the target nucleic acid molecules in an exponential manner, may theoretically have potential for single nucleic acid molecule analysis. However, nonspecific amplification background in these homogeneous reactions, that is, undesired nucleic acid amplification in the absence of the target molecule, significantly limits the sensitivity and capability of these exponential amplification methods for the detection of the nucleic acid target with low copy numbers. Therefore, up to now, despite the impressive advancement regarding ultrasensitive nucleic acid detection, the development of reliable assays that enable single-molecule nucleic acid analysis remains a big challenge.

In this contribution, by taking miRNA as a proof-of-concept nucleic acid target, we wish to report for the first time a single-microbead-based sensing (SMBS) platform, which enables the detection of miRNA at the single-molecule level by using a common fluorescence microscope. As the miRNA field continues to evolve, miRNAs have been regarded as promising diagnostic biomarkers as well as potential targets for the discovery of new anti-cancer drugs.^10^–^12^ In particular, due to the extremely low absolute number of many important miRNAs (as low as a few molecules per cell), a single-molecule miRNA assay is urgently desired in order to accurately understand the biofunction of miRNAs, miRNA-related tumor initiation and progression as well as therapeutic responses. Although the existing miRNA assays, including the methods using SMD techniques^13^,^14^ and various homogeneous nucleic acid amplification strategies,^15^–^24^ have significantly advanced the sensitivity for miRNA analysis (detection limit down to the fM to pM range), such sensitivities cannot meet the demand for single-molecule miRNA analysis in complex biosamples. In this regard, we mainly focus the application of our SMBS strategy towards the goal of single-molecule miRNA analysis in this work.

## Results and discussion

**Fig. 1:**
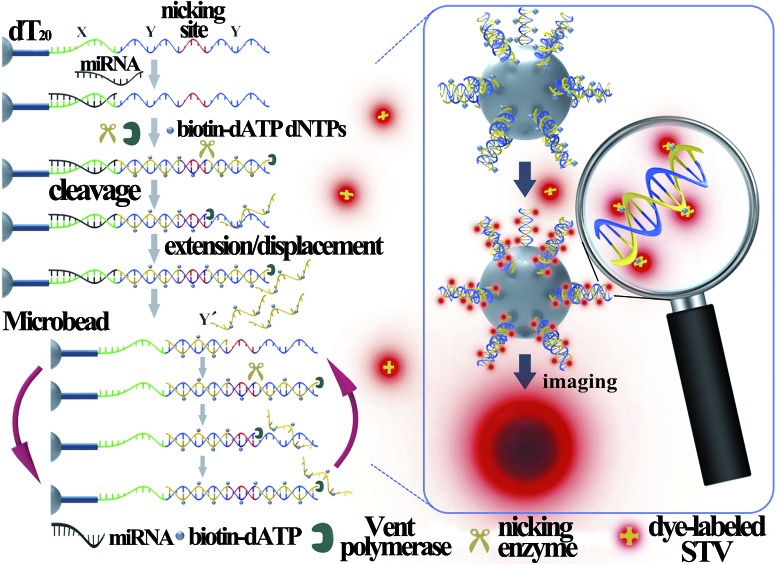
Schematic illustration of the EXPAR-SMBS system for miRNA analysis.

Fig. 1 illustrates the work principle of the SMBS-based miRNA assay, where a generic isothermal exponential amplification reaction (EXPAR) is rationally designed towards specific miRNAs and the EXPAR is conducted on the surface of a single microbead for signal amplification and fluorescence enrichment. In this newly designed EXPAR-SMBS system, a single magnetic agarose microbead, which is decorated with the EXPAR template, is used to capture the miRNA and then initiate the EXPAR on the bead surface. The X portion of the template is the anti-sense sequence of the target miRNAs, and the Y domain is designed to be a well-performing template sequence for EXPAR amplification. The sequence between the two Y sequences is a specific recognition site for the Nt.BstNBI nicking endonuclease. The oligo(dT20) at the 3′ terminus of the template is introduced as a spacer to ensure high accessibility for the target molecules and enzymes to the template anchored on the microbead surface.

In this design, the target miRNA will hybridize with its anti-sense sequence (X) at the 3′-terminus of the template and then extend along the template in the presence of Vent (exo-) DNA polymerase, dNTPs and biotinylated dATP (biotin-dATP). Afterwards, the nicking site in the middle of the two Y sequences will be specifically recognized by the nicking endonuclease Nt.BstNBI, which causes single-strand DNA nicking in the newly extended strand. The cleaved DNA strand containing the recognition site will extend again and the short single-stranded DNA (Y′) will be displaced and released due to the strand-displacement activity of Vent (exo-) DNA polymerase. In turn, the released Y′ could trigger a new extension reaction by hybridizing with the Y section of another amplification template. Thus, extension, cleavage, and strand displacement can be repeated continuously and this results in the exponential amplification under isothermal conditions. During the EXPAR, numerous biotin-dATPs will be incorporated into the EXPAR products on the microbead along with other dNTPs. As a consequence, the biotin-accumulated bead will bind with Alexa Fluor 546-labeled streptavidin (STV), which will in turn fluoresce. As such, the target miRNAs can be detected and quantified by reading the fluorescence on the microbead with a common fluorescence microscope. According to the recent study by the Niemz group,^25^ the efficiency of the EXPAR will be sequence-dependent using the traditional X–X model template. The elegantly designed X–Y–Y model template in this study will efficiently avoid the sequence-dependent amplification bias. Once the miRNAs initiate the first-step extension reaction, the subsequent enzyme-catalytic reaction would be transformed to the amplification of Y′ irrespective of the miRNA sequences, making this strategy generally applicable for assaying different miRNA species.

Although microbeads coupled with fluorescence enrichment have been previously employed for the detection of nucleic acid targets,^26^–^30^ these assays are generally performed by using thousands or even millions of small-sized beads in one reaction and accomplished by measuring the average fluorescence intensity from all the beads. When the number of copies of the nucleic acid molecules is ultralow, such as a few copies, only a few beads can generate the target-dependent fluorescence signal, which will be hidden in the average of the bulk measurement. Therefore, it is impossible to achieve detection sensitivity at the single-molecule level with these bead-based nucleic acid assays. In this work, the novel EXPAR-SMBS system utilizes a single microbead (80 ± 5 μm) as the platform to perform the miRNA amplification with the EXPAR, so all fluorescence generated from the miRNA target is enriched on only one microbead. More importantly, anchoring the EXPAR templates on a single microbead will create a microamplification zone. By pre-enriching the miRNA targets on the microbead, the unprimed amplification background^31^,^32^ can be greatly suppressed in such a small reaction zone. Therefore, it will be demonstrated that the fluorescence signal on the microbead produced by as low as 3 copies of the miRNA target can clearly be distinguished from the background because not only is the fluorescence enriched on a single microbead to greatly enhance its brightness, but also the EXPAR on the microbead surface is efficient and has a low-background signal. Moreover, monitoring the fluorescence signal of a single microbead instead of detecting the average value of a large number of microbeads should be more reliable and accurate for determining miRNA targets, especially for ultralow copy numbers of the targets.

**Fig. 2:**
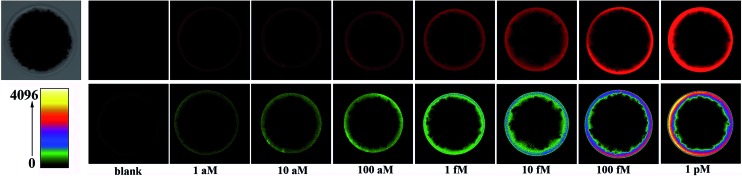
Microbead brightness as a function of let-7a concentration. Top panel: fluorescence images of the microbeads treated with ascending let-7a concentrations (under PMT HV of 450 V); bottom panel: visualization of the increasing brightness of the corresponding microbeads by using pseudocolor bars where different colors represent different fluorescence intensities.

The effect of various parameters, such as the amount of Vent (exo-) DNA polymerase and Nt.BstNBI nicking enzyme, on the performance of the EXPAR-SMBS platform has been investigated (Fig. S3 and S4, ESI†). Under the optimized conditions, the analytical performance of the EXPAR-SMBS system for let-7a miRNA detection was investigated by fluorescence imaging of the microbead upon the addition of varying concentrations of let-7a. As shown in Fig. 2, since the templates are only immobilized on the surface of the microbead, a bright halo around the bead surface is observed in the fluorescence images, the brightness of which increases gradually as the concentration of let-7a increases from 1 aM to 1 pM. If a pseudocolor bar is used to display the fluorescence image of the microbead, where different fluorescence intensities are indicated by different colors, the gradual increase of the bead brightness can be more clearly discriminated by the naked eye (bottom panel in Fig. 2). One can see from the fluorescence imaging results that a concentration as low as 1 aM (∼3 molecules in a 5 μL system) of let-7a can be clearly discerned from the blank control, demonstrating that this strategy enables let-7a analysis at the single-molecule level. To acquire the quantitative values of the fluorescent microbeads, we made a z-stack scan to cut the depth of a single microbead into 10 slices, and then the integrated fluorescence intensities of these slices were counted together to avoid possible errors from manual focusing (Fig. S2, ESI†). As shown in Fig. S5 (ESI†), let-7a can be quantitatively detected and the microbead fluorescence intensities (FI) are linearly dependent on the logarithm of the let-7a concentration in the range from 1 aM to 1 pM (0.005 zmol to 5 amol). The correlation equation is: FI = 2.68 × 10^6^ + 1.26 × 10^6^ lg *C*_miRNA_ (aM) with a correlation coefficient (*R*) of 0.9996.

**Fig. 3:**
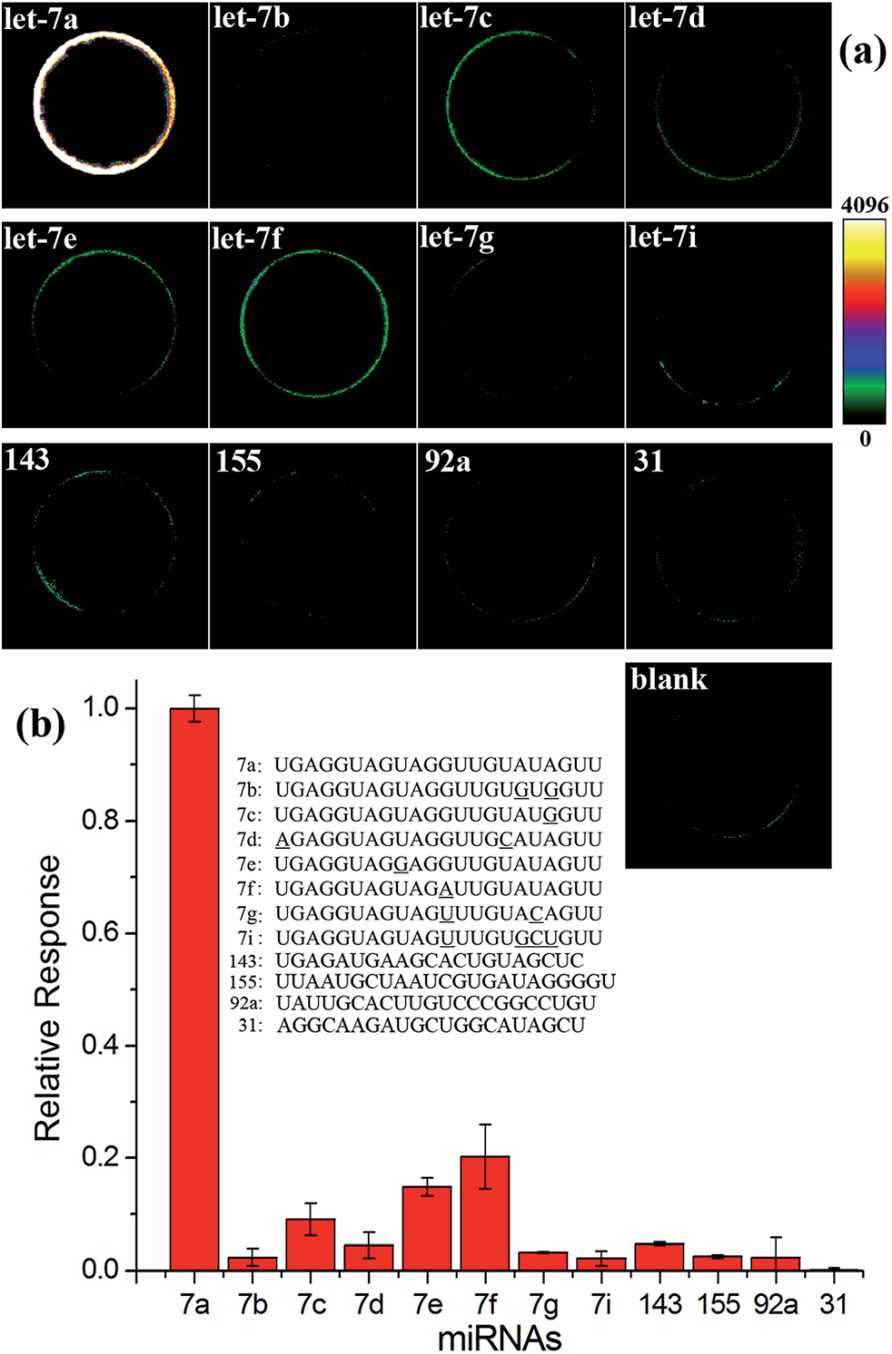
Specificity evaluation of the EXPAR-SMBS system. (a) Fluorescence images of the EXPAR-SMBS system with different miRNA targets (PMT HV for the imaging: 550 V); (b) relative responses of other miRNAs compared with that of let-7a. The concentrations of all of these miRNA targets were 10 fM.

High specificity is another important aspect to evaluate a practical miRNA assay. To interrogate the specificity of the proposed assay, the let-7a specific EXPAR-SMBS system was tested using different let-7 family members (let-7a–g and i), which have high homologous sequences with only 1–4 nucleotide differences. As can be seen from Fig. 3, only let-7a causes intense bead brightness and the other let-7 family members can be clearly discriminated from let-7a even on the basis of only one-nucleotide difference. Furthermore, other non-homologous miRNA targets only generate negligible responses. Therefore, the EXPAR-SMBS platform can be characterized with high specificity to discriminate a single-nucleotide difference among miRNA targets.

We have also testified the generality of this EXPAR-SMBS system for the detection of other miRNAs by randomly choosing mir-155 and mir-122 as model targets. The design of the EXPAR-SMBS system for mir-155 and mir-122 is the same as that for the detection of let-7a except that the sequence of the X domain in the EXPAR template is changed to be complementary to the miRNA target. As can be seen from Fig. S6 (ESI),† similar to the results from the let-7a analysis, both mir-155 and mir-122 induce a dose-responsive increase in the microbead brightness. In particular, even at a concentration as low as 1 aM, an obvious increase in the bead brightness relative to the blank control can be unequivocally detected by the naked eye for both mir-155 and mir-122, suggesting that the sequence-dependent amplification bias of EXPAR is indeed avoided by using the rationally designed X–Y–Y-type template. These results demonstrate that the EXPAR-SMBS platform is a versatile technology that could be easily extended for the detection of other miRNAs by only changing the X sequence in the EXPAR template according to the target sequence.

Further studies have been performed to test whether the EXPAR-SMBS system is feasible for the detection of let-7a in complex biological samples. The amount of let-7a in a total RNA sample extracted from HCT-116 cells was detected by this assay. As demonstrated in Fig. S7 (ESI),† with a simultaneously constructed calibration curve, the amount of let-7a in a rather low amount of total RNA (20 pg) is estimated to be 10.6 aM (in a 5 μL system). To verify the accuracy of this result, 100 aM of synthetic miRNA was spiked into the 20 pg of total RNA, and the concentration of let-7a in such a sample was determined to be 105.1 aM with a recovery of 95.5%. Therefore, this EXPAR-SMBS platform is reliable for the quantitative determination of attomolar miRNAs in complex biological matrices.

**Fig. 4:**
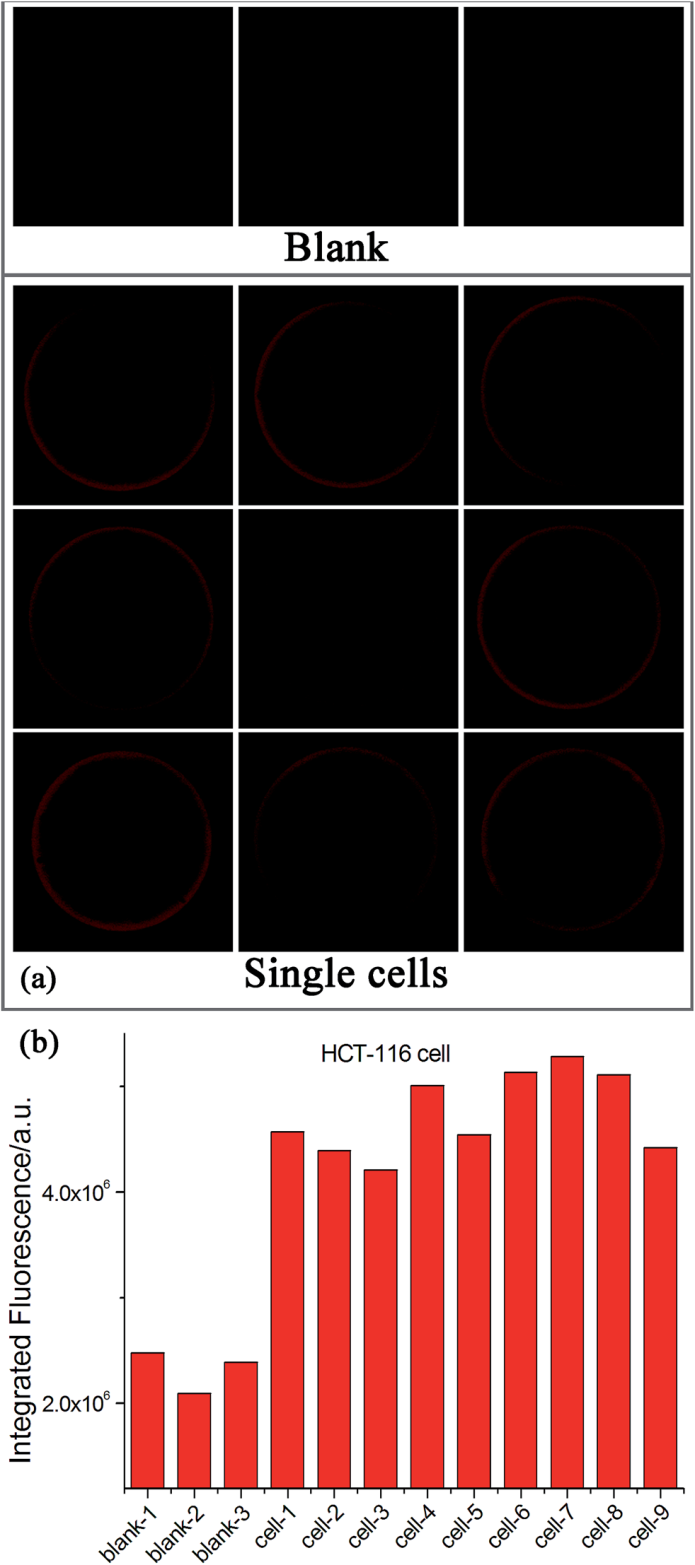
Detection of let-7a in individual HCT-116 cells. (a) Fluorescence images of the EXPAR-SMBS system with 9 single HCT-116 cells. Three blank controls (without HCT-116 cells) were also carried out simultaneously for comparison. (b) Relative responses of let-7a in these single cells.

Since many human diseases, especially cancers, often begin with cellular abnormalities in a small minority of cells within an organism, the miRNA expression can be stochastic and distinct between normal and cancer cells, and even in the same type of cells because individual cells may be at different stages.^33^–^35^ Therefore, detection of cell-to-cell variations of miRNA levels at the single-cell level is extremely important in order to provide a deep insight regarding the miRNA function as well as the exact correlation between miRNA expression and cell function, which may be masked in ensemble measurements.^33^–^36^ Due to its ultrahigh sensitivity and high tolerant capability for complex matrices, the EXPAR-SMBS system was also successfully applied for the direct detection of miRNAs in a single cell. For this study, individual HCT-116 cells were first lysed using simple heat treatment and then directly used as the sample to initiate the EXPAR on a single microbead. As illustrated in Fig. 4, even in the presence of only one cell, the increase in the fluorescence intensity of the microbead can be clearly observed relative to the blank control. The amount of let-7a determined in 9 individual cells varied from 30 copies to 350 copies. Similar variations in the number of miRNAs per cell were also observed by several other groups because individual cells may be at different stages.^33^–^35^ Furthermore, according to previous reports, the number of an individual miRNA species is roughly estimated to be in the range from dozens to several hundred per cell.^15^ Therefore, our results are reasonable and consistent with previous literature.

## Conclusions

In conclusion, we have demonstrated that the integration of the highly efficient EXPAR with fluorescence enrichment on a single microbead provides an ultrasensitive and generic miRNA assay. This protocol works with a 5 μL sample volume and requires a total assay time of only ∼40 min to accumulate enough fluorescence signals on the surface of only one microbead to allow for the unequivocal detection of the target miRNA at the single-molecule level. Due to its ultrahigh sensitivity and selectivity, this strategy is also feasible for miRNA analysis in complex biosamples even in the single cell lysate. Therefore, this robust single-molecule EXPAR-SMBS strategy may act as a powerful tool to allow for deep insight into how miRNAs influence the central signalling pathways and the cell functions at the single-cell level, which are of great significance for miRNA-related clinical studies. Furthermore, it is conceivable that the SMBS platform can also facilitate the single-molecule detection of other types of nucleic acids (*e.g.* DNA and mRNA) by combining it with appropriate nucleic acid amplification techniques.

## Supplementary Material

Supplementary information
