# Supplementary material for: Lab on a single microbead: an ultrasensitive detection strategy enabling microRNA analysis at the single-molecule level
Source: Chem Sci. 2015 Aug 20;6(11):6213–8. doi: 10.1039/c5sc02641e (PMC6054045; doi:10.1039/c5sc02641e)
Supplement: Supplementary file 1 [file SC-006-C5SC02641E-s001.pdf]

Electronic Supplementary Material (ESI) for Chemical Science.
This journal is © The Royal Society of Chemistry 2015

Electronic Supplementary Information

Lab on a Single Microbead: An Ultrasensitive Detection Strategy Enables MicroRNA Analysis at Single-Molecule Level

Xiaobo Zhang, Chenghui Liu,* Lingbo Sun, Xinrui Duan, Zhengping Li*

Key Laboratory of Applied Surface and Colloid Chemistry, Ministry of Education, Key Laboratory of Analytical Chemistry for Life Science of Shaanxi Province, School of Chemistry and Chemical Engineering, Shaanxi Normal University, Xi'an 710062, Shaanxi Province, P. R. China

List of Contents:

1. Reagents and Materials

2. Standard Protocols of the EXPAR-SMBS Platform for Let-7a Analysis

3. Optimization of the Amount of Vent (exo-) DNA Polymerase and the Nicking Enzyme

4. Quantitative Results for Detection of Let-7a MiRNA

5. Analytical Performance of the EXPAR-SMBS System for the Detection of Mir-155 and Mir-122

6. Quantitative Detection of Let-7a in Complex Cell Extracts

1. Reagents and Materials

Biotinylated EXPAR templates, all of the target miRNAs, mixture of dNTPs, RNase inhibitor and RNase-free water were purchased from TaKaRa (Dalian, China). The Vent (exo-) polymerase, Nt.BtsNBI nicking enzyme, and the ThermoPol buffer were obtained from New England BioLabs (Beverly, MA, USA). The Streptavidin Mag Sepharose microbeads, which are composed of iron oxide cores embedded in agarose matrices and are surface-functionalized with streptavidin (STV), were purchased from GE Healthcare (Uppsala, Sweden). The biotin-dATP, Alexa Fluor 546-STV conjugates and the TRIzol regent were supplied by Life Technologies (Carlsbad, CA, USA). All of other chemical reagents were of analytical grade and used without further purification.

The sequences used in let-7a-specific EXPAR-SMBS system are listed below (5′→3′):

Let-7a: UGAGGUAGUAGGUUGUAUAGUU

Template: AACTATCGACAACTTC(Y)-AACAGACTCA(nicking site)- AACTATCGACAACTTC (Y)-AACTATACAACCTACTACCTCA(X, anti-miRNA)-TTTTTTTTTTTTTTTTTTTT(T20 spacer)-biotin

2. Standard Protocols of the EXPAR-SMBS Platform for Let-7a Analysis

Conjugation of the EXPAR templates on the microbeads. After thoroughly blending, 10 μL slurry of the Streptavidin Mag Sepharose microbeads (STV-beads) was pipetted into a 1.5 mL centrifuge tube and washed twice with TBS buffer (50 mM Tris-HCl, 150 mM NaCl, pH 7.5) via magnetic isolation. Afterward, an excess amount of biotinylated EXPAR template (1 nmol) was incubated with the STV-beads for 30 min under shaking. Then these microbeads were magnetically separated to remove the

excess unbound EXPAR template and washed three times with RNase-free water. Finally, the template-conjugated microbeads were re-suspended in 500 μL of RNase-free water and ready for subsequent use.

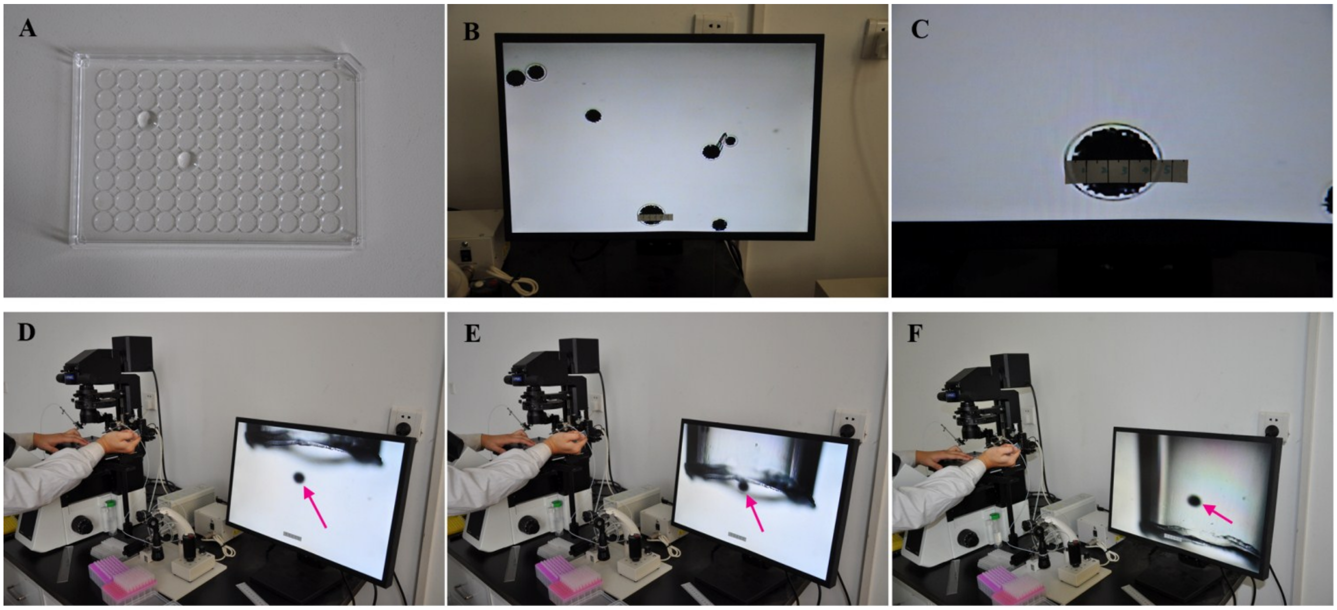

Fig. S1. Illustration of the catching process of a single magnetic microbead.

Manipulation of a single microbead. Fig. S1 illustrates the catching process of a single microbead. A hydrophobic 96-well plate cover with transparent property can keep about 100 μL water (containing 2 μL of EXPAR template-conjugated beads) in the shallow wells (image A), which is an ideal platform to assist the capture of individual microbeads. A Narishige micromanipulator system equipped on an Olympus IX53 inverted microscope with a monitor is used to manipulate a single microbead (image B~F). With the help of the camera and monitor, one can search the suitable microbeads on the screen by naked eyes (image B). A DIY ruler (image C) is pasted on the screen to help us select the beads with the desired narrow size (80 ± 5 μM). Once the desirable microbead is selected from the screen, we only need a pipette to catch it (in 1 μL volume, image D~F) and the manipulation of a single microbead only needs 1~2 min.

Standard EXPAR-SMBS procedures. The reaction mixtures for the EXPAR-SMBS system were prepared separately on ice as part A and part B. Part A consisted of 0.5× Nt.BstNBI buffer, 0.5× ThermoPol buffer, RNase inhibitor and the miRNA target. Part B consisted of 0.5× ThermoPol buffer, dNTPs, biotin-dATP, Nt.BstNBI nicking endonuclease, Vent (exo$^-$) DNA polymerase, and RNase-free water.

Typically, a single template-conjugated microbead was firstly incubated with 3 μL of mixture A under vigorous shaking at 45ºC for 1 hour to enrich the target miRNA on the bead surface. Then, the part B was added to form a totally 5 μL of EXPAR-SMBS reaction system containing biotin-dATP (8 μM), dATP (8 μM), dTTP (16 μM), dCTP (16 μM), dGTP (16 μM), Nt.BstNBI (0.3 U/μL), Vent (exo$^-$) DNA polymerase (0.02 U/μL), RNase inhibitor (0.8 U/μL), 1×ThermoPol buffer (20 mM Tris–HCl, pH 8.8, 10 mM KCl, 10 mM $(NH_4)_2SO_4$, 2 mM $MgSO_4$, 0.1% Triton X-100), and 0.5× Nt.BstNBI buffer (25 mM Tris–HCl, pH 7.9, 50 mM NaCl, 5 mM $MgCl_2$, 0.5 mM dithiothreitol). The EXPAR-SMBS reaction was performed at 55ºC for 38 min (T100 Thermal Cycler, BIO-RAD). After the EXPAR-SMBS reaction, the single microbead was washed by PBS buffer (20 mM phosphate buffer containing 300 mM NaCl) and followed by the addition of 5 μL of excess Alexa Fluor 546-STV (50 μg/mL) to stain the biotin-dATP incorporated in the EXPAR products on the microbead surface. After a further cycle of washing and isolation steps, the bead was immediately subjected to fluorescence imaging.

Moreover, for the single-cell let-7a analysis, a single HCT-116 cell was picked up by a capillary (diameter of 100 μm) with the micromanipulator system (Narishige, equipped on an Olympus IX53 microscope). Then the single cell was suspended into 1 μL of RNase-free water and lysed at 95ºC for 5 min, which was immediately used as the sample to perform the EXPAR-SMBS reaction according to

the standard procedures stated above.

Fluorescence imaging test. All images were taken using an Olympus FV-1200 laser scanning confocal microscope. The microbead in 5 μL of PBS buffer was spread on a cover slip and the fluorescence image was obtained by collecting the fluorescence at the wavelength range from 565 nm to 665 nm under the excitation of a 559 nm laser. In particular, in order to acquire the quantitative value of a fluorescent microbead for quantitative analysis of miRNAs, we made a z-stack scan to cut the depth of a single microbead into 10 slices (see illustration in Fig. S2), and the integrated fluorescence intensities of these slices were counted together to avoid the possible errors during manual focusing.

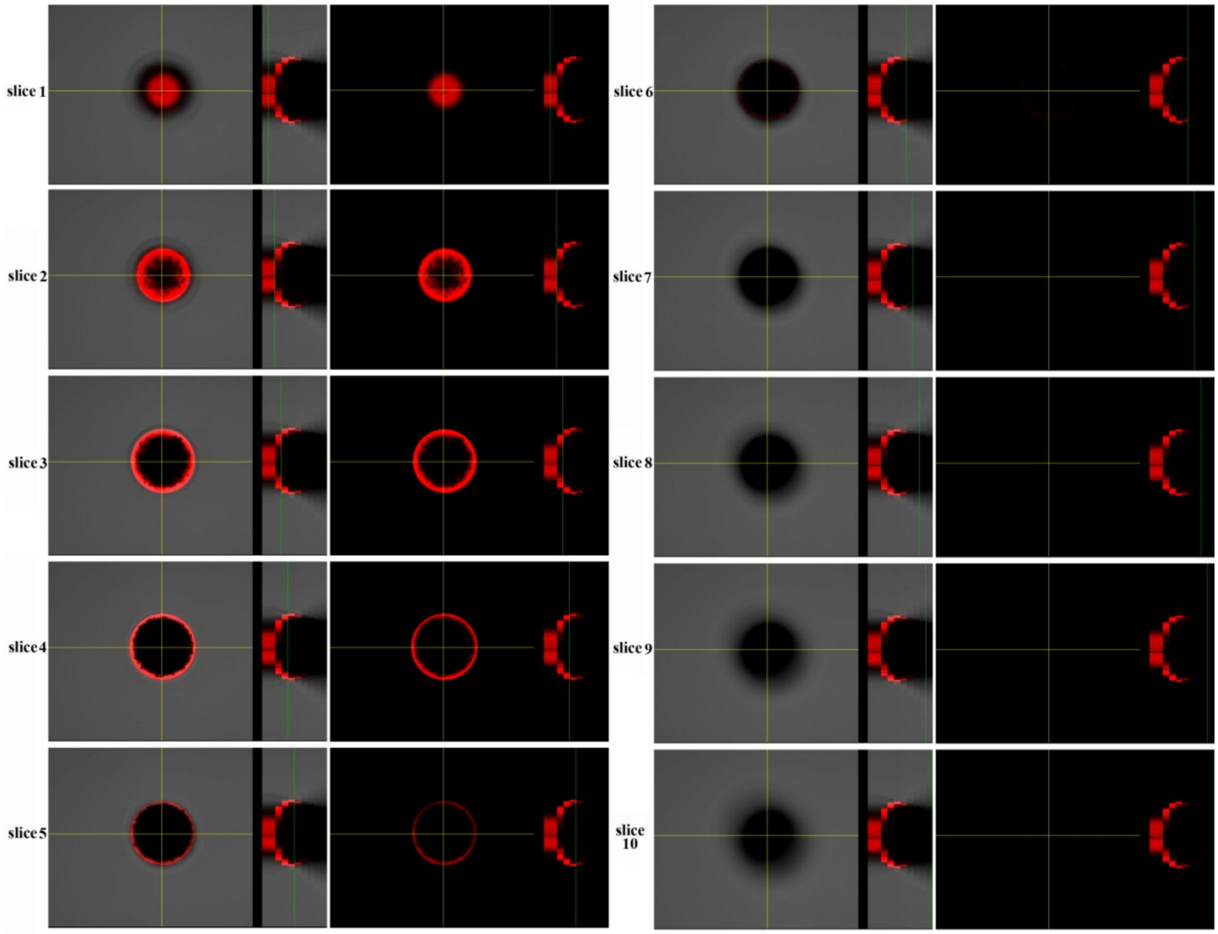

Fig. S2. Schematic illustration of the laser confocal fluorescence scanning mode (z-stack) by cutting a

single microbead into 10 slices. For each slice, the right panel is the fluorescence image and the left panel is the bright field image merged with the fluorescence image.

It also should be noted that for the quantitative analysis of miRNAs, the maximum fluorescence value of a bright spot that can be quantitatively acquired by the fluorescence microscope is 4096. In order to acquire brighter microbead image but not exceed this maximum value, the PMT HV of the fluorescence microscope may be reasonably tuned for the imaging of microbeads at different batches, and the used PMT HV values are provided in the corresponding figure captions in the manuscript.

3. Optimization of the Amount of Vent (exo-) DNA Polymerase and the Nicking Enzyme

According to the principle of EXPAR, the amplification efficiency will be seriously influenced by the amount of DNA polymerase and nicking enzyme. The effect of the amount of Vent (exo^-) polymerase was first investigated in this study. The integrated fluorescence signal of the microbead treated by 10 fM let-7a was recorded respectively by using 0.01 U/μL, 0.015 U/μL, 0.02 U/μL, 0.03 U/μL Vent (exo^-) DNA polymerase. The blank was treated under the same procedures without let-7a. It can be seen from Fig. S3 that with the increase of Vent (exo^-) polymerase, the reaction time, at which the fluorescence signals produced by 10 fM let-7a can be clearly discriminated from the blank, is gradually shortened. In consideration of all of these parameters such as high microbead brightness, high S/B ratio and short assay time, 0.02 U/μL Vent (exo^-) DNA polymerase with a 42 min reaction time was selected to be the optimum.

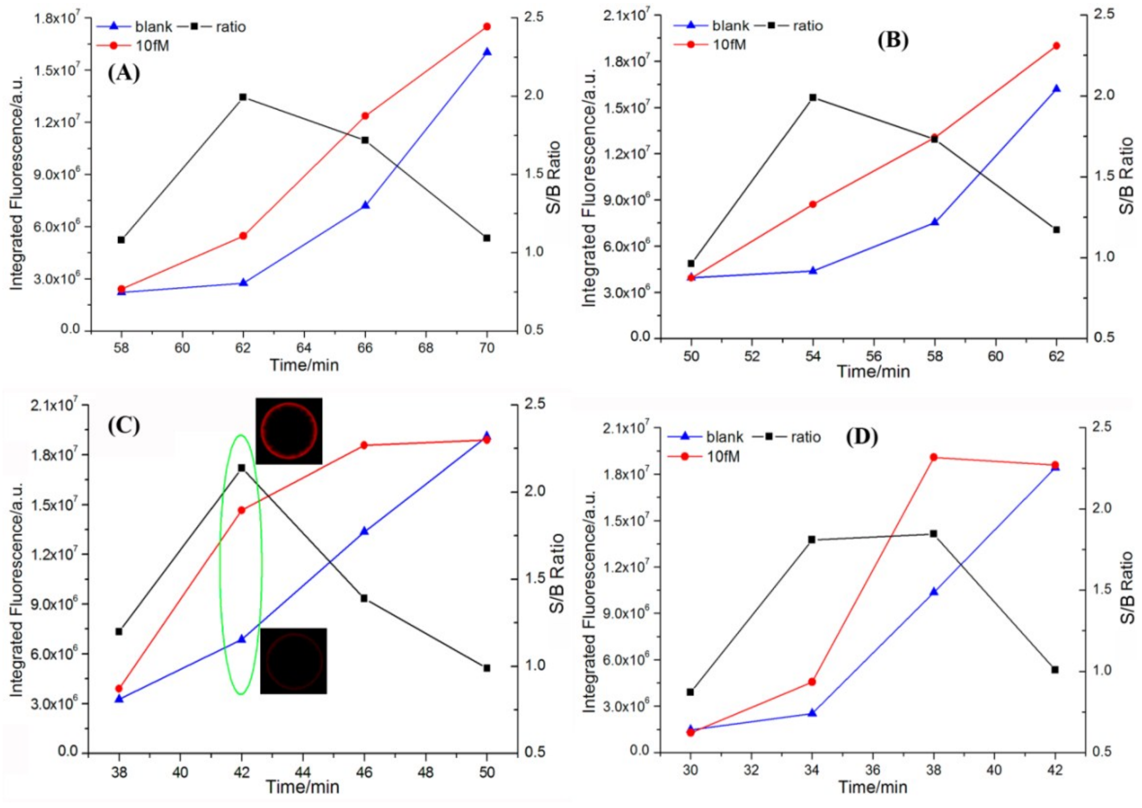

Fig. S3. Effect of the amount of Vent (exo$^-$) DNA polymerase. The integrated fluorescence intensities of individual microbeads treated with 10 fM let-7a and the blank control under the catalysis of varying concentrations of Vent (exo$^-$) were recorded respectively at different times. The amount of Vent (exo-): (A) 0.01 U/μL; (B) 0.015 U/μL; (C) 0.02 U/μL; (D) 0.03 U/μL. The amount of Nt.BtsNBI nicking enzyme was fixed at 0.4 U/μL. The S/B ratio refers to the ratio of bead signal produced by 10 fM let-7a to the blank signal.

Furthermore, the amount of Nt.BtsNBI nicking enzyme was further optimized by fixing Vent (exo$^-$) DNA polymerase at 0.02 U/μL, and the results were shown in Fig. S4. One can see that at Nt.BtsNBI enzyme concentration of 0.3 U/μL, the ratio of microbead signal produced by 10 fM let-7a to the blank signal (S/B ratio) can be further obviously enhanced at a shorter assay time of 38 min. Therefore, 0.3 U/μL Nt.BtsNBI nicking enzyme coupled with 0.02 U/μL Vent (exo$^-$) DNA polymerase with a reaction

time of 38 min were subsequently used as the optimal EXPAR-SMBS conditions for the detection of miRNAs.

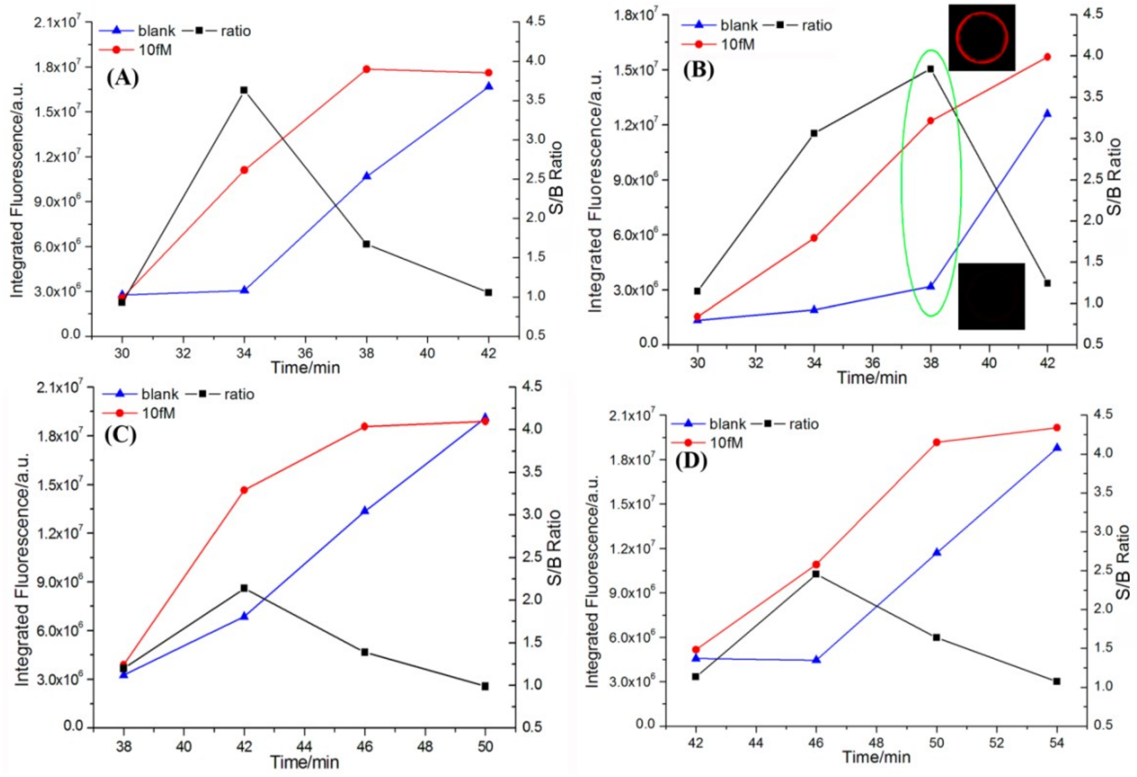

Fig. S4. The influence of the amount of Nt.BtsNBI nicking enzyme on the performance of EXPAR-SMBS system. The integrated fluorescence intensities of individual microbead treated with 10 fM let-7a and the blank control were recorded respectively in the presence of varying concentrations of Nt.BtsNBI enzyme at different times. The amount of Nt.BtsNBI nicking enzyme in this study: (A) 0.2 U/μL; (B) 0.3 U/μL; (C) 0.4 U/μL; (D) 0.5 U/μL. The amount of Vent (exo-) polymerase was fixed at 0.02 U/μL.

4. Quantitative Results for Detection of Let-7a MiRNA.

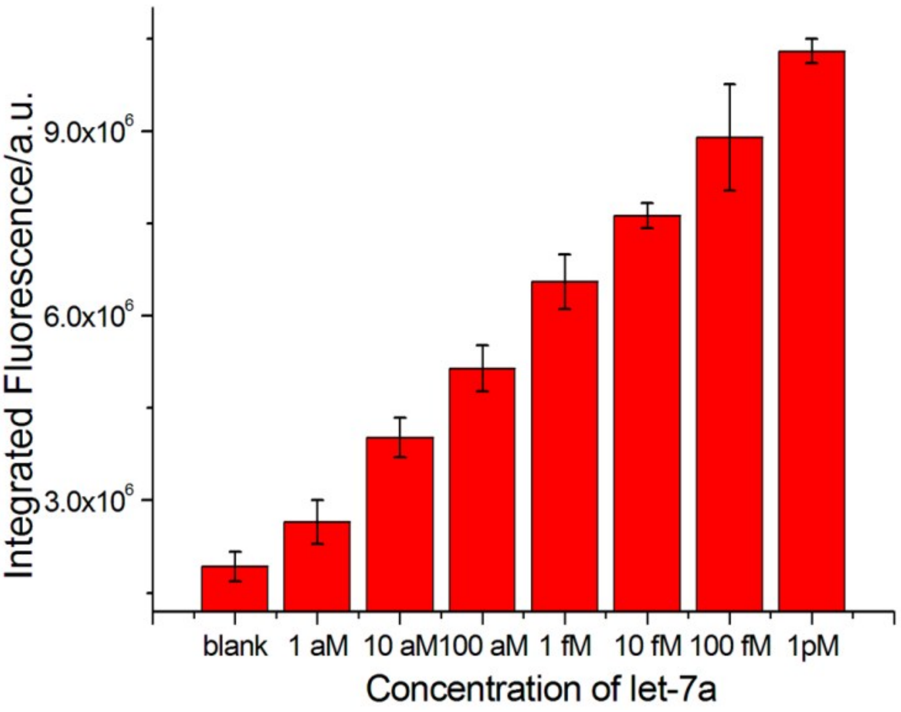

Fig. S5. Plot of the integrated fluorescence signal of the microbead as a function of let-7a concentration. Error bars represents the standard deviation from three repetitive measurements.

5. Analytical Performance of the EXPAR-SMBS System for the Detection of mir-155 and mir-122

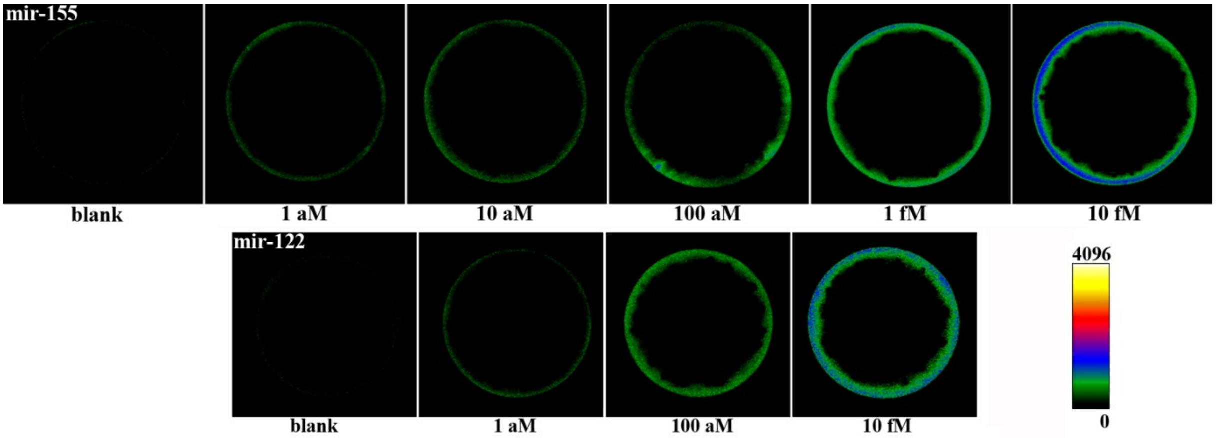

Fig. S6. Analytical performance of the EXPAR-SMBS strategy for the detection of mir-155 (top panel) and mir-122 (bottom panel). PMT HV for the imaging: 450 V. The EXPAR-SMBS reaction for both mir-122 and mir-155 were performed according to the same experimental conditions as those for let-7a

without further optimization.

6. Quantitative Detection of Let-7a in Complex Cell Extracts

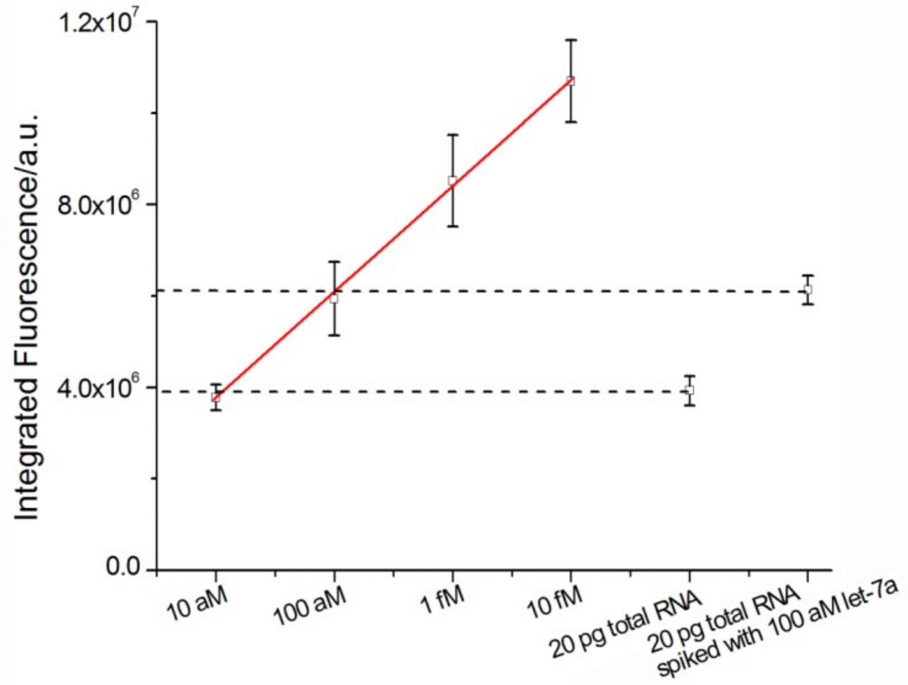

Fig. S7. Quantitative detection of let-7a in complex cell extracts by using a simultaneously constructed calibration curve.
